# Population genetic analysis based on the polymorphisms mediated by transposons in the genomes of pig

**DOI:** 10.1093/dnares/dsae008

**Published:** 2024-03-06

**Authors:** Wencheng Zong, Runze Zhao, Xiaoyan Wang, Chenyu Zhou, Jinbu Wang, Cai Chen, Naiqi Niu, Yao Zheng, Li Chen, Xin Liu, Xinhua Hou, Fuping Zhao, Ligang Wang, Lixian Wang, Chengyi Song, Longchao Zhang

**Affiliations:** State Key Laboratory of Animal Biotech Breeding, Institute of Animal Sciences, Chinese Academy of Agricultural Sciences (CAAS), Beijing, China; State Key Laboratory of Animal Biotech Breeding, Institute of Animal Sciences, Chinese Academy of Agricultural Sciences (CAAS), Beijing, China; College of Animal Science, Shanxi Agricultural University, Jinzhong, China; College of Animal Science and Technology, Yangzhou University, Yangzhou, China; College of Animal Science and Technology, Yangzhou University, Yangzhou, China; State Key Laboratory of Animal Biotech Breeding, Institute of Animal Sciences, Chinese Academy of Agricultural Sciences (CAAS), Beijing, China; College of Animal Science and Technology, Yangzhou University, Yangzhou, China; State Key Laboratory of Animal Biotech Breeding, Institute of Animal Sciences, Chinese Academy of Agricultural Sciences (CAAS), Beijing, China; College of Animal Science and Technology, Yangzhou University, Yangzhou, China; State Key Laboratory of Animal Biotech Breeding, Institute of Animal Sciences, Chinese Academy of Agricultural Sciences (CAAS), Beijing, China; Chongqing Academy of Animal Science, Chongqing, China; State Key Laboratory of Animal Biotech Breeding, Institute of Animal Sciences, Chinese Academy of Agricultural Sciences (CAAS), Beijing, China; State Key Laboratory of Animal Biotech Breeding, Institute of Animal Sciences, Chinese Academy of Agricultural Sciences (CAAS), Beijing, China; State Key Laboratory of Animal Biotech Breeding, Institute of Animal Sciences, Chinese Academy of Agricultural Sciences (CAAS), Beijing, China; State Key Laboratory of Animal Biotech Breeding, Institute of Animal Sciences, Chinese Academy of Agricultural Sciences (CAAS), Beijing, China; State Key Laboratory of Animal Biotech Breeding, Institute of Animal Sciences, Chinese Academy of Agricultural Sciences (CAAS), Beijing, China; College of Animal Science and Technology, Yangzhou University, Yangzhou, China; State Key Laboratory of Animal Biotech Breeding, Institute of Animal Sciences, Chinese Academy of Agricultural Sciences (CAAS), Beijing, China

**Keywords:** pig, transposable elements, structural variations, population genetic, transduction

## Abstract

Transposable elements (TEs) mobility is capable of generating a large number of structural variants (SVs), which can have considerable potential as molecular markers for genetic analysis and molecular breeding in livestock. Our results showed that the pig genome contains mainly TE-SVs generated by short interspersed nuclear elements (51,873/76.49%), followed by long interspersed nuclear elements (11,131/16.41%), and more than 84% of the common TE-SVs (Minor allele frequency, MAF > 0.10) were validated to be polymorphic. Subsequently, we utilized the identified TE-SVs to gain insights into the population structure, resulting in clear differentiation among the three pig groups and facilitating the identification of relationships within Chinese local pig breeds. In addition, we investigated the frequencies of TEs in the gene coding regions of different pig groups and annotated the respective TE types, related genes, and functional pathways. Through genome-wide comparisons of Large White pigs and Chinese local pigs utilizing the Beijing Black pigs, we identified TE-mediated SVs associated with quantitative trait loci and observed that they were mainly involved in carcass traits and meat quality traits. Lastly, we present the first documented evidence of TE transduction in the pig genome.

## 1. Introduction

Transposable elements (TEs), also known as mobile elements or transposons, comprise approximately half of the mammalian genome.^1,2^ The transposition of TEs can result in diverse genetic effects, such as genomic instability, which has been extensively reported in humans and is associated with the development of diseases.^3^ In addition, TEs mediate structural variations (SVs), including insertions or deletions, in the genome. These SVs have been demonstrated to drive genome evolution, increase population diversity, alter gene transcription, and cause phenotypic variations.^4–7^ TEs are classified into retrotransposons and DNA transposons based on their transposition mechanism. Retrotransposons can be classified into three main categories: long interspersed nuclear elements (LINEs), short interspersed nuclear elements (SINEs), and long terminal repeats (LTRs).^8^ Previous studies have reported that LINEs account for 18.52% of the pig genome, LTRs account for 7.56%, and SINEs account for 11.05%.^9^ SVs mediated by TEs are currently considered a distinct type of genetic variation, separate from general insertions or deletions.^10^ TEs are characterized by the presence of functional elements.^11^

The pig (*Sus scrofa*) is an important agricultural animal that was domesticated approximately 9,000 years ago in Anatolia and the Mekong Valley.^12,13^ Through long-term natural and artificial selection, successful commercial pig breeds such as the Large White, Landrace, and Duroc pigs have been developed to exhibit high growth rates, high lean meat ratios, and high feed efficiency. However, these breeds often exhibit reduced intramuscular fat content. In China, various pig breeds retain their unique ancestral traits. For example, the Meishan pig is known for its high reproduction rate, the Laiwu pig has a high intramuscular fat content, and the Min pig and Rongchang pig exhibit exceptional environmental adaptability.^14^ In addition, there are excellent crossbred pig breeds, such as the Beijing Black pig, which have multiple domestic and foreign blood sources and possess desirable traits from both commercial and Chinese local pig breeds.^15,16^

Although TEs have been extensively annotated in the pig genome and their genetic impacts have been studied,^9^ there is currently a lack of large-scale mining of TE-SVs using resequencing genome data in pigs. In addition, 20–30 retrotransposon insertion polymorphisms (RIPs) identified from the assembled pig genomes were previously utilized for population genetic analyses and their association with economic traits was investigated.^17–20^ In this study, we conducted a comprehensive mining of TE-SVs using genome sequencing data from nine Chinese local pig breeds, one commercial pig breed, and one crossbred pig breed. By utilizing these TE-SVs, we reconstructed the population structure of 11 pig breeds. We thoroughly investigated the distribution of all TE-SVs in pig genomes and annotated all loci associated with PTVs. Additionally, Fst scanning was performed to identify candidate divergence regions (CDRs) of the crossbred pigs with Chinese local and commercial pigs. The results indicate that these CDRs coincide with specific Quantitative Trait Loci (QTL) traits unique to different pig groups. Furthermore, we conducted the first investigation of L1 3ʹ transduction in the pig genome and identified three loci that may be related to variations in protein-coding genes.

## 2. Materials and methods

### 2.1 Sample collection and sequencing

The ear tissues of 35 Beijing Black pigs (BJB) used in this experiment were provided by Beijing Heiliu Animal Husbandry Technology Co., Ltd. (Beijing, China). DNA was extracted from the ear tissues using the phenol–chloroform method. The concentration and quality of DNA were assessed using a NANODROP 1000 spectrophotometer (Thermo Scientific, Waltham, MA, USA). Paired-end sequencing was performed on the Illumina HiSeq 2500 platform, which generated a total of approximately 252G of sequencing data (zipped) from 35 Beijing Black pigs. The QC metrics for the data are provided in Supplementary Table S1. The sequencing data of 35 Beijing Black pigs used in this study have been submitted to the National Center for Biotechnology Information (NCBI) with the accession number PRJNA1077662. The resequencing data from a previous study included data from 30 Large White pigs (LW), 7 Min pigs (M), 3 Lantang pigs (LT), 3 Diannan small-ear pigs (DN), 3 Bama pigs (BM), 4 Meishan pigs (MS), 3 Neijiang pigs (NJ), 4 Tongcheng pigs (TC), 4 Nanyang pigs (NY), and 5 Mashen pigs (MaS). All relevant information about the resequencing data is provided in Supplementary Table S2.

### 2.2 Data processing

Trimmomatic^21^ was used to trim the raw reads, removing adapter sequences and low-quality reads. The high-quality trimmed reads were then aligned against the pig reference genome (Sscrofa11.1) using Bwa software.^22^ Samtools^23^ was used to convert sam files to bam format, followed by sorting and indexing the bam files. PCR duplicates were marked using Picard.^24^ Read-depth statistics of all samples were calculated using mosdepth.^25^

### 2.3 TE-SVs calling

MELT^26^ was used to identify TEs in the pig genome. The MELT-SPLIT and MELT-Deletion programs were used to identify TE-SV insertions and deletions, respectively. All TE-SVs were subsequently filtered based on the following criteria: (i) deletion of loci smaller than 50 bp; (ii) retention of loci with ‘PASS’; (iii) evidence of at least one-side target site duplication (TSD) for insertions. TEs were further classified using the Repbase database (https://www.girinst.org/).

### 2.4 TE-SVs detection

A total of 12 pig breeds were utilized for TE-SVs genotyping via PCR. The breeds included three commercial breeds (Duroc, Landrace, and Large white pigs from Anhui Province), seven native breeds (Erhualian, Fengjin, and Jiangquhai pigs from Jiangsu Province, Wuzhishan pigs from Hainan Province, Bama pigs from Guangxi, Ningxiang pigs from Hunan Province, and Huai pigs from Fujian Province), and two crossbreeds (Sujiang and Sushan pigs from Jiangsu Province). For each breed, three individual DNA samples were combined for PCR detection. In total, 30 SINEs, 70 LINEs, and 40 LTRs (MAF > 0.10) were randomly sampled from the genomes for primer design (see schematic shown in Supplementary Fig. S1 and primer sequences listed in Supplementary Table S3) and subsequent PCR detection.

### 2.5 Population structure reconstruction

Principal component analysis (PCA) was carried out using GCTA.^27^ Phylip^28^ was employed to construct the NJ tree, and the tree was subsequently processed and visualized using MEGA11.^29^ Admixture^30^ was used to carry out the evaluation of the population structure and to calculate the possible groupings (*K* = 3 and 11).

### 2.6 Annotation of TE-SVs and analysis of PTVs

The locations of all TE-SVs were annotated using gene location information from the Ensembl database (https://useast.ensembl.org/Sus_scrofa/Info/Index/). Genotypes were visualized using the ggplot2 package^31^ via R. Kyoto Encyclopaedia of Genes and Genomes (KEGG) functional enrichment analysis was performed using the kobas online website (http://kobas.cbi.pku.edu.cn/genelist/), and pathways with *P*-values < 0.05 were retained.

### 2.7 F_ST_ scanning

Vcftools^32^ was used to calculate F_ST_ in sliding 500-kb windows with 50-kb steps along the autosomes. Comparisons were made between crossbred and Chinese local pig breeds, as well as between crossbred and commercial pig breeds.

### 2.8 L1 3ʹ transduction screening

LINE-1 (L1) 3ʹ transduction was detected using the MELT’s Transduction Find pipeline. The resulting VCF was manually analysed to extract the METRANS and MESOURCE fields, allowing for the identification of the transduction location.

## 3. Result

### 3.1 Genome-wide TE-SVs mining in pig

In this study, we conducted re-sequencing of the crossbreed (Beijing Black Pig), followed by the retrieval of SRA data from the commercial breed (Large White Pig) and nine Chinese local pig breeds (Bama, Diannan Small-Ear, Lantang, Mashen, Meishan, Min, Nanyang, Neijiang, and Tongcheng pigs) with the aim of identifying TE-SVs. The whole genome sequencing data of 101 pig samples, with an average coverage of 15.03 × (Supplementary Tables S1 and S2), were mapped to the pig reference genome (Sscrofa11.1) using the bwa-mem algorithm. The resulting alignments were converted to bam files, which were then sorted and marked to identify duplicate reads. Subsequently, we used the MELT software to detect TE insertions and deletions and performed genotyping.

**Figure 1.**
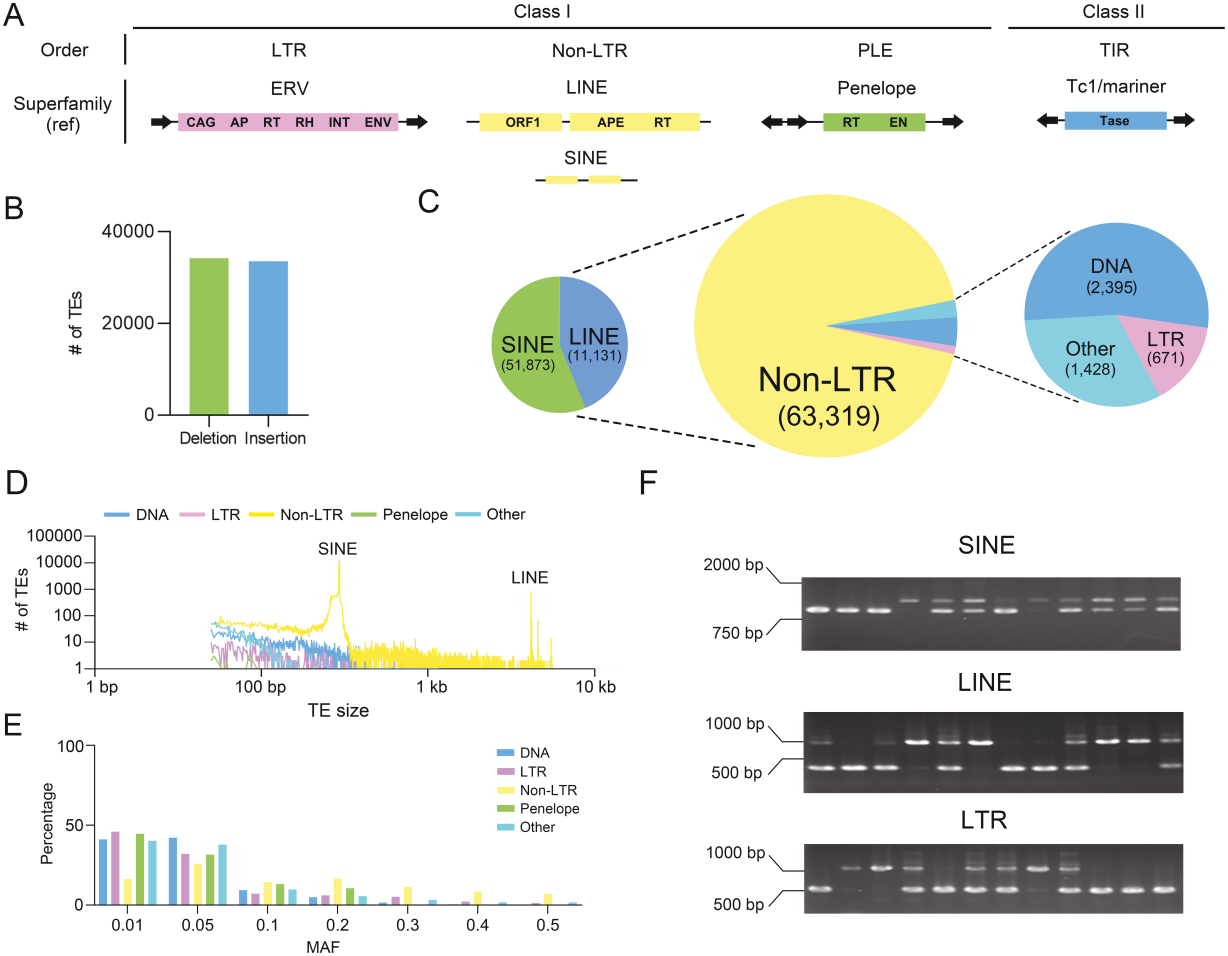
Polymorphic TE map of the pig genome. (A) Representation of TE orders and superfamilies in this study. (B) Number of TE insertions and deletions. The bar graph represents the number of insertions and deletions in the final TE dataset. (C) TE classification percentages. The central pie chart depicts the distribution of TE types, with the left chart highlighting the major TE types in the No-LTR group, and the right chart indicating the percentage of TE types other than non-LTRs. The Arabic numerals indicate the count of each TE type. (D) TE size distribution per TE type with *x*-axis and *y*-axis shown in log_10_ scale. (E) Distribution of TE based on MAF. The *x*-axis represents the distribution of MAF intervals (starting from zero), while the *y*-axis represents the percentage of different TE types within each interval. (F) Representative electrophoretic assay graphs showing polymorphic loci. SINE1, LINE47, and LTR33 were chosen as representative examples to showcase polymorphism in pooled DNA samples from 12 pig breeds.

In total, we identified 67,813 TE-SVs that passed our standard quality control as described in the methods (Fig. 1A and Supplementary Fig. S2). The number of identified insertion and deletion alleles was nearly equal (Fig. 1B). Retrotransposons accounted for the majority of these TE loci (94.36%), with SINE being the most abundant (51,873/76.49%), followed by LINE (11,131/16.41%). LTR retrotransposons, DNA transposons, and other TEs constituted a smaller proportion, totalling 4,494 loci (Fig. 1C). We analysed the length distribution of all TE-SVs (Fig. 1D) and found that the majority (90.70%) were less than or equal to 500 bp. This is because SINEs contain small fragments (≤500 bp), and almost half (44.17%) of LINE-mediated TE-SVs also fall into this size range (Supplementary Fig. S3 and Supplementary Table S4). These results suggest that the host genome tolerates the insertion of small fragment retrotransposons. Further analysis revealed that the majority of the identified TE-SVs are rare alleles (MAF < 0.10), accounting for 58.85% (39,905) of the total TE-SVs (Fig. 1E and Supplementary Table S5). The common TE-SVs were genotyped by PCR using DNA samples from 12 domesticated pig breeds (MAF > 0.10), which have the potential to be used in future genetic analyses, and loci for which genotypes could not be determined will be excluded from the statistics. Over 84% of the TE-SVs were found to be polymorphic (Fig. 1F, Supplementary Fig. S4 and Supplementary Table S6), indicating the high reliability of the obtained TE-SVs.

### 3.2 Recovering population genetic structure using TEs

**Figure 2.**
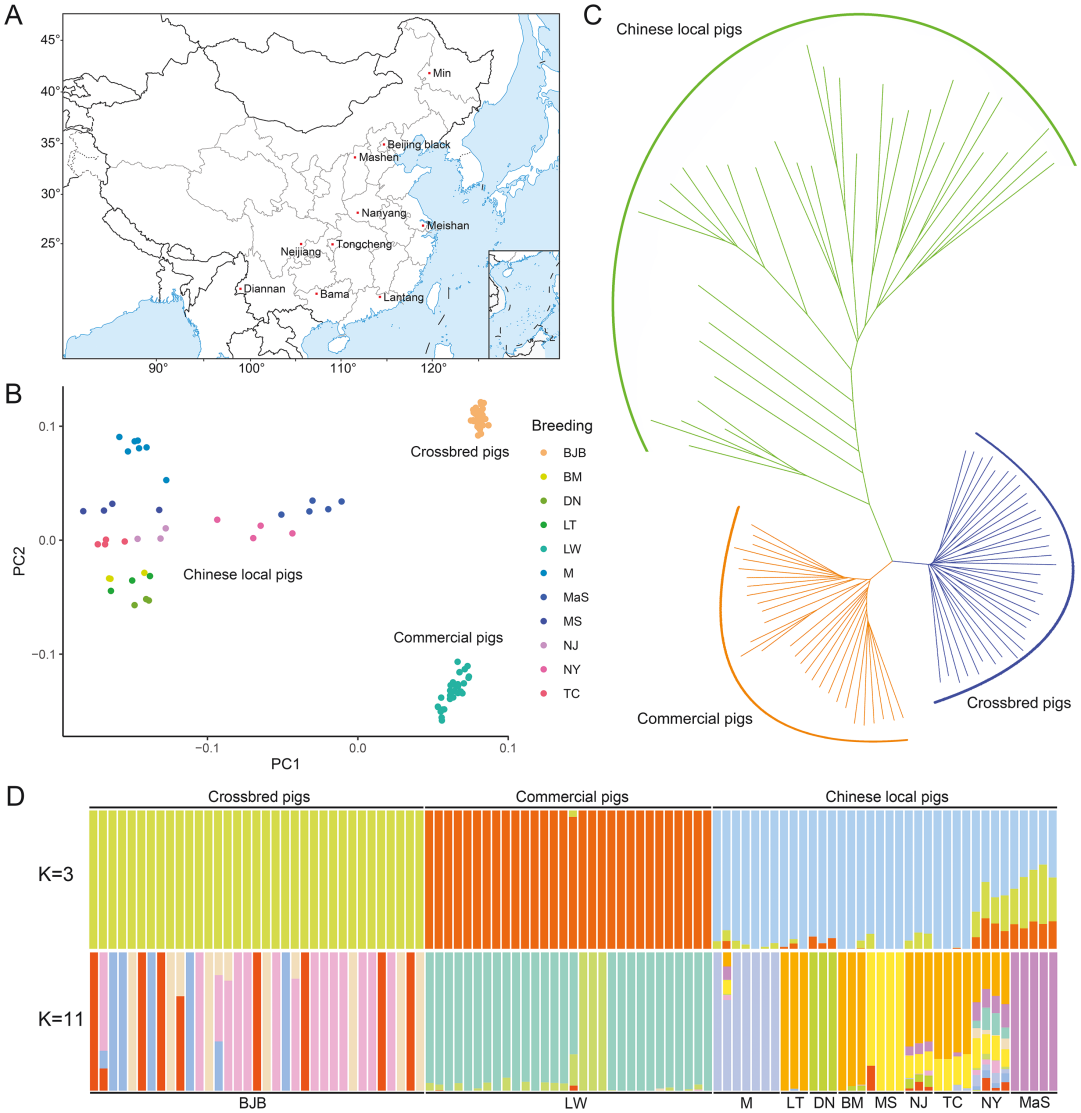
Population genetic analysis utilizing TEs. (A) Geographical distribution of the Chinese pig breeds included in this study. (B) Principal component analysis. All individuals are plotted according to their coordinates on the biplot of PC1 versus PC2. Each colour represents a different breed, and the abbreviations for the breeds are provided on the right side of the chart. (C) The phylogenetic tree was constructed based on all the individuals in this study. The eleven pig breeds were categorized into three groups, representing Chinese local, commercial, and crossbred pigs. (D) Genome-wide admixture analyses inferred from TEs (*K* = 3 and 11). The top portion of the figure displays the segregation of the 11 breeds into three groups, representing Chinese local, commercial, and crossbred pigs. The bottom portion illustrates the specific groupings for each breed. Each individual is depicted as a vertical rectangle with distinct colours, indicating different genetic populations. Sample name Abbreviations: Beijing Black pig, BJB; Large White pig, LW; Min pig, M; Lantang pig, LT; Diannan small-ear pig, DN; Bama pig, BM; Meishan pig, MS; Neijiang pig, NJ; Tongcheng pig, TC; Nanyang pig, NY; Mashen pig, MaS.

We conducted a population structure analysis to validate the accuracy of the identified TE-SVs. Prior to the analysis, we filtered all the obtained TE-SVs using plink with the following parameters: --geno 0.1 --maf 0.05 --chr 1-18. Subsequently, we used a total of 31,208 TE-SVs for the population genetics analysis (Fig. 2A–D). PCA separated nine Chinese local pig breeds, one commercial pig breed, and one crossbred pig breed into three distinct groups (Fig. 2B). Additionally, we constructed a phylogenetic tree, which revealed that Chinese local, commercial, and crossbred pigs formed three separate clusters (Fig. 2C and Supplementary Fig. S5). To investigate the ancestral lineage of each pig breed, we performed a population structure analysis using admixture at two values of *K*: *K* = 3 and *K* = 11, which corresponded to assumed breed pedigree distributions (Fig. 2D). The results indicated that the 101 pigs were clearly divided into three populations at *K* = 3, aligning with the results from the PCA and phylogenetic tree analyses. At *K* = 11, Beijing Black pigs exhibited the presence of four different pedigree sources, suggesting a complex pedigree during their breeding. Among the local pig breeds in China, Min, Diannan small-ear, Meishan, and Mashen pigs displayed distinct pedigrees. Southern Chinese pig breeds (Bama, Lantang, Neijiang, and Tongcheng pigs) exhibited relatively more similar pedigree compositions compared with northern pigs (Min and Mashen pigs). Lantang and Bama pigs shared a similar genetic background, likely due to their similar characteristics as small-sized pigs and their geographical proximity. In contrast, Diannan small-ear pigs, being located near the border region, may have experienced restricted gene flow across a wider area.

### 3.3 Impact of TE-SVs on genes

**Figure 3.**
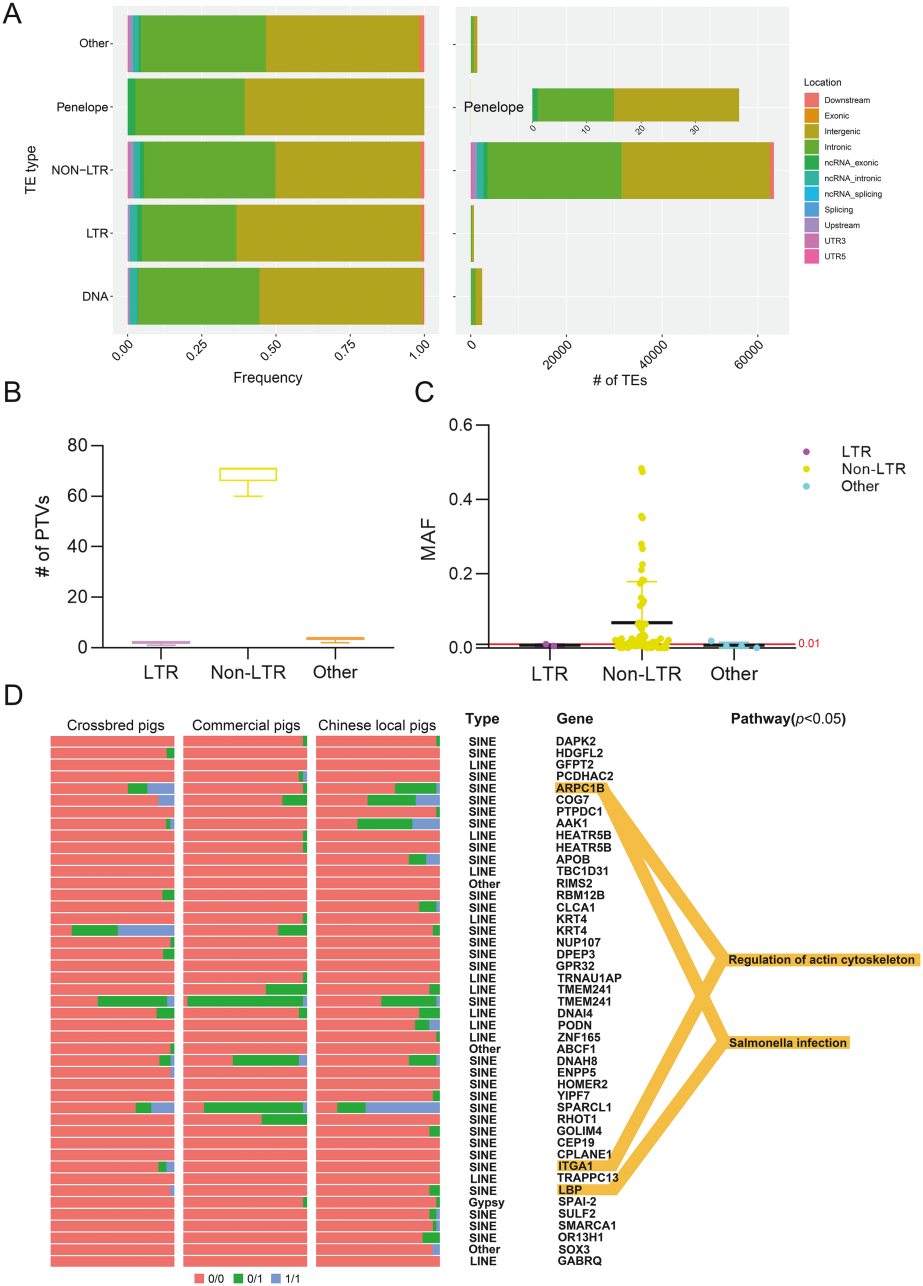
TE functional analysis. (A) Distribution of different TE loci in the pig genome. The left plot displays the percentage of each TE type at different genomic locations, while the right plot shows the number of each TE type at different genomic locations. (B) Number of PTVs. Box plots displaying the count of PTVs categorized by LTR, Non-LTR, and other TE types for all individuals. (C) MAF distribution of PTVs. The scatter plot illustrates the MAF distribution of PTVs, classifying PTVs with MAFs below 0.01 as private variants. (D) PTVs genotype visualization and pathway analysis. The plot classifies the 11 pig breeds as Chinese local, commercial, and crossbred pigs, displaying the genotypes for the 45 PTV loci associated with the encoded genes. Additionally, the plot includes TE type annotations for each PTV, along with their corresponding coding genes. The pathway analysis results for the 42 coding genes with a significance level of *P* < 0.05 are shown on the right side.

To assess the functional impact of TE-SVs, we annotated the distribution of TE-SVs in the genome based on gene information from the Ensemble database. The majority of TE-SVs (93.30%) were located in intergenic and intronic regions, while only 6.70% were found in other regions (Fig. 3A). TE insertions in coding regions can potentially disrupt open-reading frames, resulting in the loss of gene function. Therefore, we investigated the potential protein truncation variants (PTVs) generated by TE mobility, which are the coding regions of protein-coding genes containing TE-SVs. The analysis identified a total of 77 PTVs, with an average of 1.98 LTRs, 68.79 non-LTRs, and 3.48 other TE types per genome (Fig. 3B). Among these, 27 PTVs were found to be private TEs (MAF < 0.01) (Fig. 3C). Additionally, we examined the genotype frequencies of all PTVs in coding genes to identify potential functional loci in crossbred, commercial, and Chinese local pig breeds. Among the coding genes, 42 of them had overlaps with a total of 45 PTVs, with the major TE types being LINE and SINE (Fig. 3D). Following this, we performed KEGG analysis on all genes harbouring PTVs and identified two significant pathways (*P* < 0.05): one associated with the regulation of actin cytoskeleton and the other related to Salmonella infection (Fig. 3D). These findings suggest a potential connection between these pathways and breed-specific motility, meat quality, and disease resistance.

### 3.4 Population selection scanning reveal candidate adaptive traits of Beijing Black pigs by utilizing TE-SVs

**Figure 4.**
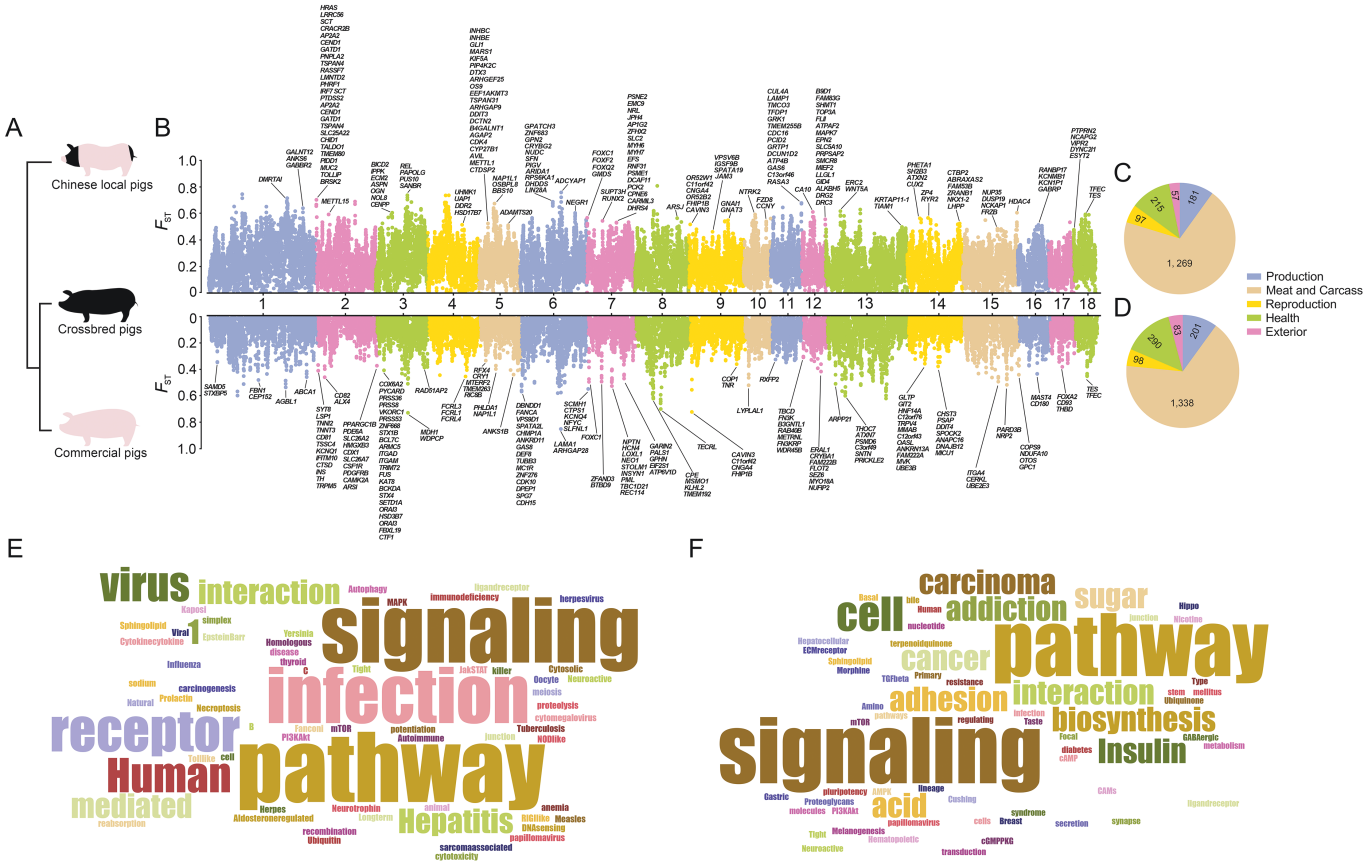
Chromosome divergence region scanning of the pig genome. (A) Phylogenetic relationships of the three pig groups in this study. (B) F_ST_ scanning between Chinese local and crossbred pigs as well as between commercial and crossbred pigs. All significantly peaked CDRs are selected and annotated for encoding genes in this region. (C–D) QTL traits overlapping with CDR peaks between (C) Chinese local and crossbred pigs as well as between (D) commercial and crossbred pigs. (E–F) Arabic numbers represent the number of QTL traits. KEGG enrichment analysis of the top 1% CDR (E) between Chinese local and crossbred pigs as well as (F) between commercial and crossbred pigs. Pathways with a *P*-value < 0.05 were retained.

TEs have emerged as reliable molecular markers in plant and animal breeding research.^33–36^ Therefore, we used TEs to conduct genome-wide scans on various pig groups. To identify specific genomic regions that differentiate crossbred pig breed from both commercial and Chinese local pig breeds, we performed F_ST_ analyses based on TE-SVs. This analysis utilized a window size of 500-kb and steps of 50-kb (Fig. 4A and B). We annotated the TE-SVs within each significant peak that overlapped with coding genes. To determine the traits associated with these regions, we utilized the pig QTL database. All identified regions of divergence were found to overlap with five categories of traits: production, meat and carcass, reproduction, health, and exterior traits. Notably, there were 1,819 regions corresponding to 302 traits that were shared between crossbred and Chinese local pigs (Fig. 4C). In contrast, there were 2,010 regions encompassing 305 traits that were shared between crossbred and commercial pigs (Fig. 4D). We further analysed the overlapping traits between these two groups using Venn plots (Supplementary Fig. S6). This analysis revealed 29 traits unique to Chinese local and crossbred pigs, as well as 32 traits unique to commercial and crossbred pigs, highlighting group-specific traits identified during the selection process (Supplementary Fig. S6 and Supplementary Table S7). A comparison between crossbred and Chinese local pigs indicated variations in body size, as suggested by carcass traits such as biceps brachii length, half carcass weight, rib weight, and backfat line at the last rib. On the other hand, differences in motility and external appearance were suggested by time spent walking and ear size when comparing crossbred pigs to commercial pigs. Variations in reproductive performance were indicated by uterine horn length, while potential meat quality differences were suggested by mean glycolytic potential, number of white fibres, and lactate/white fibre diameter; Variations in body size were reflected in loin muscle width, loin weight, shoulder weight, and carcass width. Potential associations with immunity were indicated by eosinophil number, phagocytic activity, IgM positive leukocyte number, and granular leukocyte to lymphocyte ratio. To gain a comprehensive understanding of the function of the identified regions of divergence, we conducted KEGG enrichment analysis using the top 1% of these regions. Pathways linked to regions of divergence between crossbred and Chinese local pigs showed enrichment in environmental information processing, human diseases, cellular processes, genetic information processing, and organismal systems primarily related to the immune system (*P* < 0.05) (Fig. 4E and Supplementary Table S8). In contrast, pathways associated with regions of differentiation between crossbred and commercial pigs showed enrichment (*P* < 0.05) in metabolic processes related to cofactor and vitamin metabolism, lipid metabolism, and carbohydrate metabolism, as well as in organismal systems such as endocrine, sensory, immune, digestive, and nervous systems, except in the environmental information processing, human diseases, and cellular processes (Fig. 4F and Supplementary Table S9). Based on our study, we conclude that the genomic regions of divergence in Beijing Black pigs, when compared with commercial and Chinese local pig breeds, are strongly associated with carcass traits and meat quality traits. These findings provide insights into the selection and breeding process of the Beijing Black Pig.

### 3.5 L1 3ʹ transduction in the pig genome

**Figure 5.**
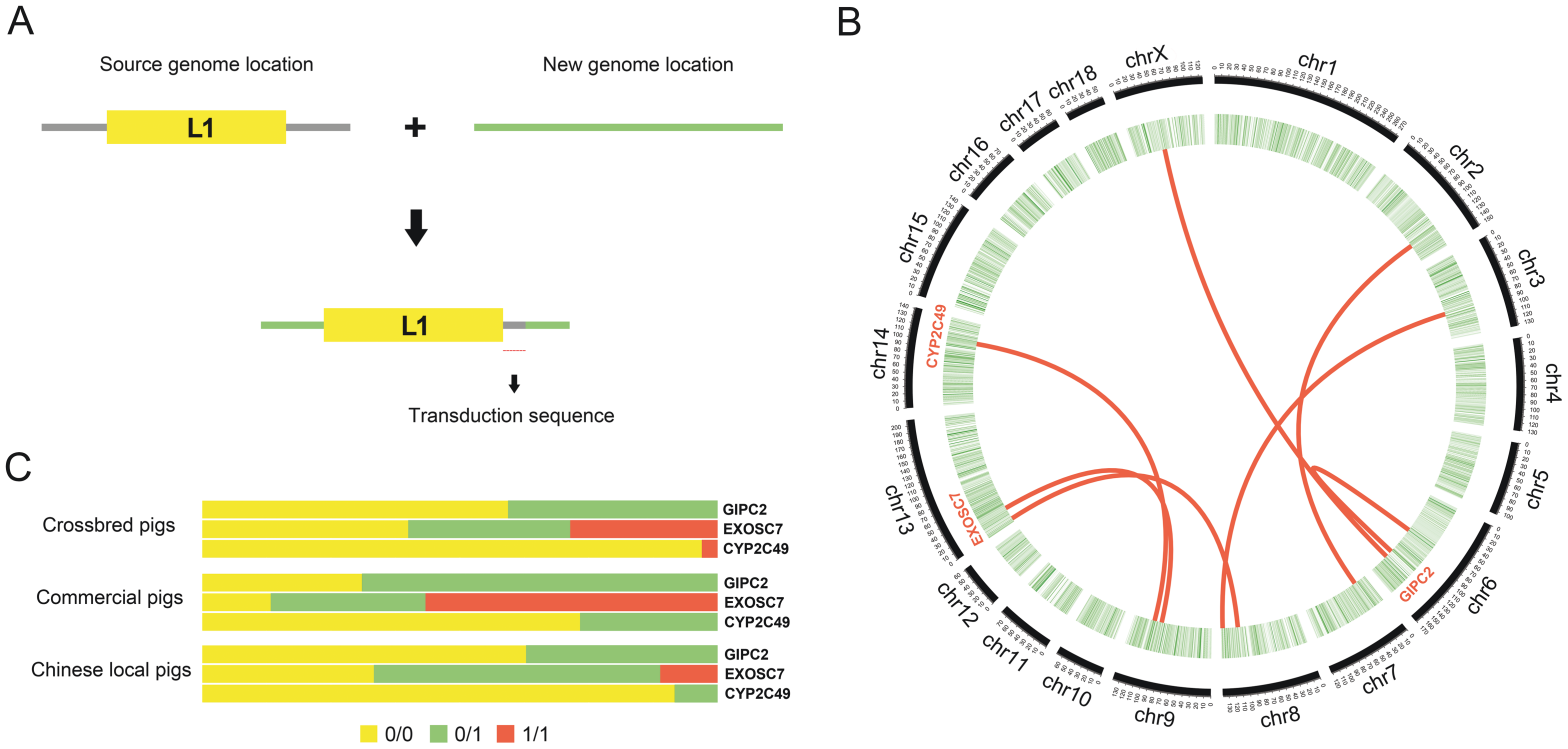
L1 3ʹ transduction analysis. (A) Diagram of the transduction mechanism. The diagram illustrates how the L1 3ʹ transduction process transfers the source genomic location sequence to a new location. (B) The circos diagram demonstrates regions of the genome undergoing transduction. Coding gene regions that undergo L1 3ʹ transduction are marked on the diagram. (C) Genotype frequencies of three transduction-associated genes. We visualized the genotype frequencies of TE-SVs located in three gene regions in Chinese local, commercial, and crossbred pig groups.

L1 possess the capacity to modify the genome through three mechanisms: insertion, rearrangement, and transduction. Active L1 elements are able to relocate non-L1 DNA sequences located at their 3ʹ ends to a new genomic location, a process known as transduction^37^ (Fig. 5A). Transduction occurs during the transcription of the L1 element, where termination is not mediated by the original poly A signal but by a stronger downstream poly A signal. In this study, we conducted an extensive examination of L1 3ʹ transduction, utilizing all identified L1 loci. We predicted a total of seven loci capable of transduction, including one intrachromosomal transduction and six interchromosomal transductions (Fig. 5B and Supplementary Table S10). These seven transduction loci involve three coding genes located in the intronic regions of *GIPC2*, *EXOSC7*, and *CYP2C49*. Additionally, we investigated the genotype frequencies of the L1 transduction loci in these three genes across different pig populations. We observed genotype frequency differences in all three loci among the three pig groups (Fig. 5C). In conclusion, this study represents the initial report on L1 3ʹ transduction in the pig genome; however, further validation is required to determine the impact of transduction on gene expression and whether specific genotypes are associated with phenotypic traits.

## 4. Discussion

As a parasitic mobile DNA element, TE plays a significant role in the pig genome. TE-based genetic markers are valuable tools for studying genetic diversity, evolution, and genetics and breeding research in plants and animals.^18,33–36,38,39^ Nonetheless, there remains a scarcity of resources for exploring TEs at a population level in the pig genome, particularly when compared with the available resources for SNVs and SVs. In this study, a multi-breed polymorphic TE map was generated utilizing one crossbred pig breed, one commercial pig breed, and nine Chinese local pig breeds. We annotated and verified the polymorphism rates of all types of TEs at the population scale, confirming the reliability of each locus. We suggest establishing reliable TE maps for each domesticated animal and conducting genetic breeding studies focusing on the potential influence of TE through dose effects on specific traits, an area gaining increasing attention in plant genome research.

TEs have a significant impact on insertional mutations during genome transmission, particularly when they occur within exons, which frequently results in disruption.^40^ We detected PTVs in 42 coding genes, typically indicating modified gene function. Among these genes, *PCDHAC2* is involved in the regulation of synaptic transmission and the generation of specific synaptic connections.^41^*SPARCL1* not only promotes synapse formation in rodent and human neurons,^42–44^ but also selectively enhances excitatory synaptogenesis and synaptic transmission through a novel mechanism independent of neuropilin and neural ligands.^45^ Duplication of the *SOX3* gene results in neural tube defects.^46^*SMARCA1* plays a crucial role in the maturation of midbrain dopaminergic (mDA) neurons^47^ and has been associated with multiple mechanisms of neurogenesis and schizophrenia.^48^*ARPC1B* is an essential component of the *ARP2/3* complex that regulates steady-state signalling in immune cells.^49^ Loss of function of *ITGAL* increases susceptibility to *Salmonella typhimurium* infection.^50^*LBP* contributes to the immune response to gram-positive bacterial and fungal infections.^51–53^ While the direct relationship between the *COG7* gene and lipid metabolism function has not been described, members of the COG complex are known to be involved in intra-Golgi trafficking and glycosylation of proteins and lipids.^54^*APOB* has been reported to play a crucial role in lipid transport and energy metabolism,^55^ and blood *APOB* levels are closely associated with growth-related traits in animals.^56–58^ Additionally, mutations in *DNAH8* lead to multiple morphological abnormalities in sperm flagella and male infertility.^59^*KRT4* is closely associated with hair growth and desmosome composition in pigs.^60^*CLCA1* is associated with mucus production in respiratory and intestinal sites.^61^ Knockout of *AAK1* can alleviate neuropathic pain.^62^ We propose the need for additional identification and comprehension of the contribution of specific genes and variants to quantitative trait variation, as well as the development and incorporation of advantageous alleles through genome editing for enhancing animal breeding programs. Importantly, although PTVs hold significant research potential, these loci necessitate additional validation due to the requirement of a safety margin in identifying the exact genomic coordinates of the variants.^63^

As widely acknowledged, the lineage of the Beijing Black pig stems from diverse origins, encompassing Northern Chinese pig breeds, Southern Chinese pig breeds, Huanghuaihai pigs, Large White pigs, Berkshire, and others. Beijing Black pig meat is highly favoured by consumers in northern China for its exceptional quality and taste. Previous studies on Beijing Black pigs have focused on using the Beijing Black pig population to perform GWAS to uncover candidate genes for vertebral and teat number,^64^ rib number,^65^ intramuscular fat content,^66^ loin muscle area,^67^ and backfat quality^68^ trait associations. Moreover, the analysis of selection signals using Beijing Black pigs and commercial pig breeds has exposed distinctive phenotypic characteristics associated with meat quality, reproduction, and immune processes.^15^ To gain insight into the direction of the Beijing Black pig, we conducted a comparative analysis of its genome-wide CDRs with those of native and commercial pigs. Our findings revealed that the main divergence between the Beijing Black pig and other pig breeds was associated with carcass characteristics, meat quality, appearance, and health traits. This study provides further confirmation that TE-based whole-gene scanning of CDRs aligns with the findings of previous research, thus affirming the dependability of TE markers and their application as breeding markers.

Transduction, as one of the primary mechanisms of L1 insertion, is a relatively common phenomenon in the human genome,^69–73^ but has not been observed in the pig genome. Under specific conditions, transduction events have the ability to widely disperse exons, genes, and regulatory elements across the genome. In contrast to the thousands of L1 transduction events observed in the human genome,^74,75^ we identified only seven transactivation events in the pig genome, three of which were located within the coding gene region. Among these genes, *GIPC2* has been identified as a candidate gene for villus hair traits in pigs^76^ and exhibits higher expression during the resting phase of hair follicles compared with the anagen phase.^77^ Although limited research reports are available on *EXOSC7*, the *EXOSC* gene family is recognized as a molecular marker for mantle cell lymphoma (MCL).^78^ Moreover, *CYP2C49* polymorphisms could be utilized to reduce boar taint levels.^79^ Previous studies have reported frequent occurrences of somatic L1 3’ transduction during dynamic tumour evolution.^74^ However, due to pigs being primarily bred for commercial purposes, the majority of the pigs used for sequencing were healthy individuals, which could explain the low occurrence of L1 3ʹ transduction in this study.

## 5. Conclusion

In this study, we conducted a comprehensive analysis of genome-wide TE-SVs in multiple pig breeds using re-sequencing data. Our analysis led to the discovery of over 65,000 TE-SVs, with more than 40% of them classified as common variants (MAF > 0.10). These TE-SVs demonstrated reliable polymorphisms. Furthermore, we utilized these genetic loci to perform population genetics analysis, which allowed us to characterize the variations in body size and meat quality between Beijing Black pigs and other commercial as well as Chinese local pig breeds. In conclusion, our study establishes a solid theoretical foundation for TE-SVs as a novel molecular marker in future research and strengthens the potential for genetic analysis as well as molecular breeding in livestock, including pigs.

## Supplementary Material

dsae008_suppl_Supplementary_Table

dsae008_suppl_Supplementary_Figure

## Data Availability

The sequencing data of 35 Beijing Black pigs used in this study have been submitted to the National Center for Biotechnology Information (NCBI) with the accession number PRJNA1077662.
