# Supplementary material for: Population genetic analysis based on the polymorphisms mediated by transposons in the genomes of pig
Source: DNA Res. 2024 Mar 6;31(2):dsae008. doi: 10.1093/dnares/dsae008 (PMC11090087; doi:10.1093/dnares/dsae008)
Supplement: dsae008_suppl_Supplementary_Figure [file dsae008_suppl_supplementary_figure.docx]

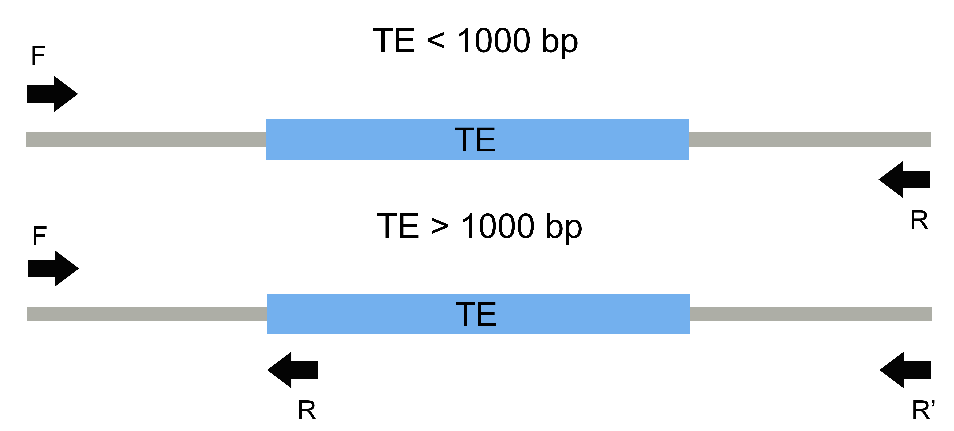


Fig S1. Primer design schematic. Two primer pairs were designed to detect polymorphism in LINE sequences larger than 1,000 bp.





Fig S2. Distribution of identified TEs in the pig genome. The circular diagram illustrates the chromosomal distribution of TEs, with concentric circles representing the following categories from outermost to innermost: DNA, LTR, Non-LTR, Penelope, and Other TE types.


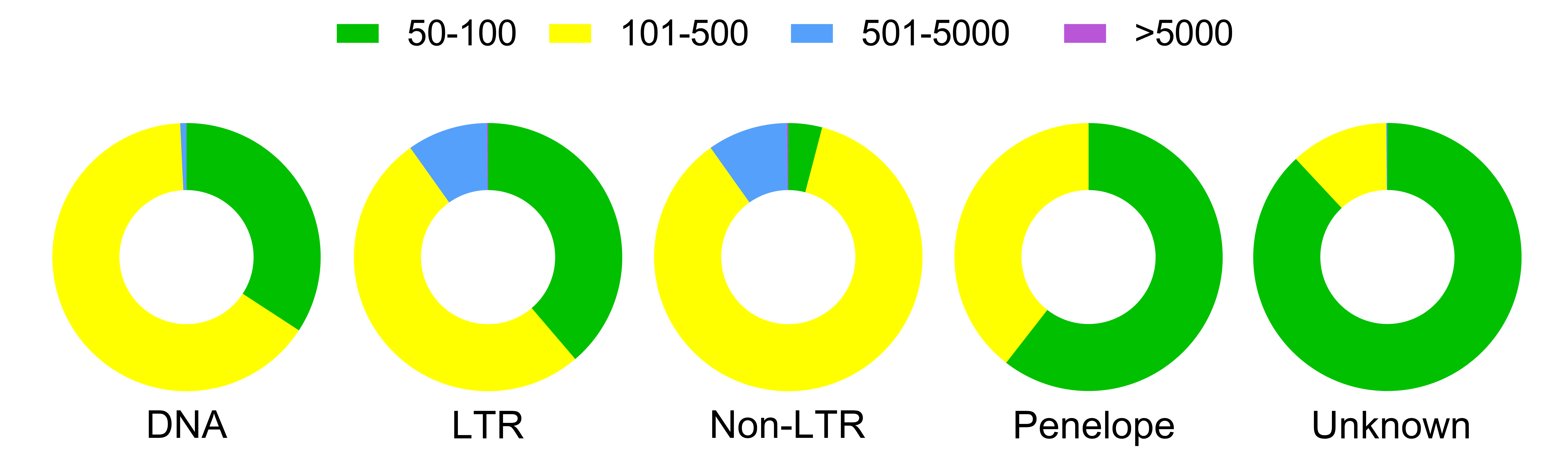


Fig S3. Distribution of length range per TE type. Above the doughnut chart, four length ranges are labeled.


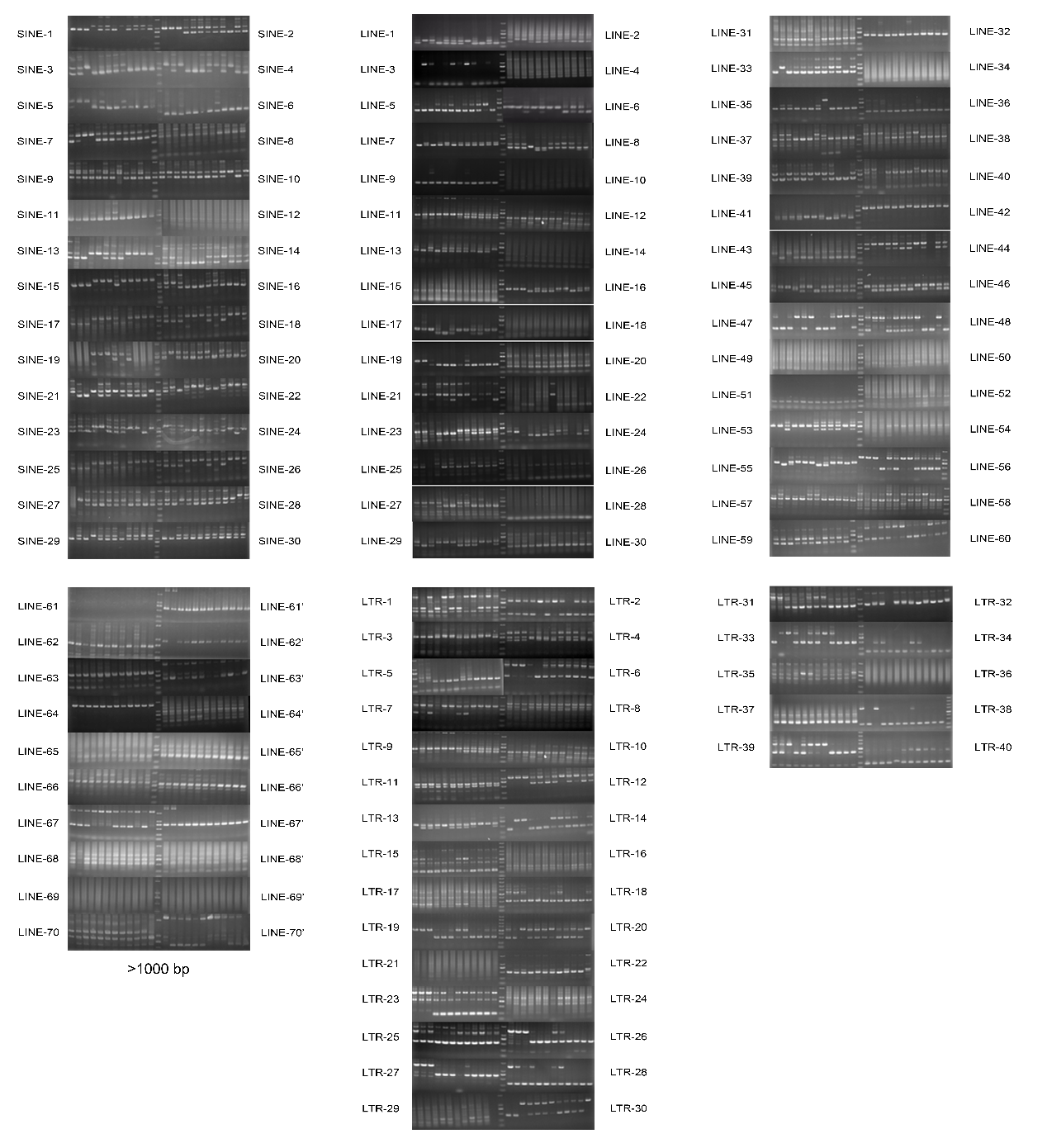


Fig S4. Electropherograms were generated to detect polymorphisms. A randomized set comprising 30 SINEs, 70 LINEs, and 40 LTRs sequences was employed for validation. Electrophoresis was performed on pig breeds including Duroc, Landrace, Yorkshire, Erhualian, Fengjin, Jiangquhai, Sujiang, Sushan, Wuzhishan, Bama, Ningxiang, and Huai pigs. Three individuals are included in each electric pool. Takara DL2000 and DL5000 markers were used to indicate the electrophoretic bands.





Fig S5. Uncollapsed phylogenetic tree was constructed based on TE data from all individuals. The phylogenetic tree was inferred using the Phylip based on the neighbor-joining (NJ) method.


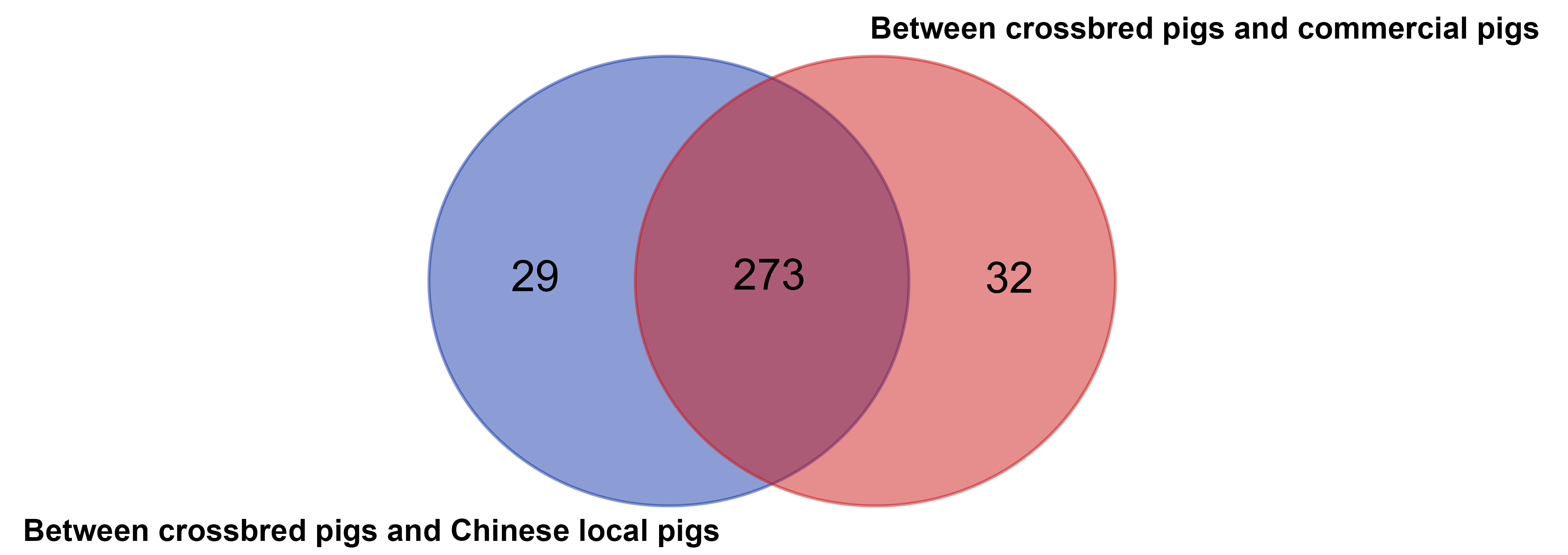


Fig S6. Venn diagrams revealed 32 private traits between crossbred and Chinese local pigs as well as 29 private traits between crossbred and commercial pigs.
